# Gemcitabine plus vinorelbine in advanced non-small cell lung cancer: a phase II study of three different doses

**DOI:** 10.1054/bjoc.2000.1341

**Published:** 2000-08-17

**Authors:** C Gridelli, L Frontini, F Perrone, C Gallo, M Gulisano, S Cigolari, F Castiglione, S F Robbiati, G Gasparini, G P Ianniello, A Farris, M C Locatelli, R Felletti, E Piazza, on behalf of the Gem Vin Investigators, See Appendix for complete list of Authors and Institutions

**Keywords:** gemcitabine, vinorelbine, non-small cell lung cancer

## Abstract

Our aim was to study the activity and toxicity of the gemcitabine plus vinorelbine (Gem Vin) combination and to identify the optimal dose. Previously untreated patients aged < 70 years, with stage IV or IIIb (not candidates for radiotherapy) non-small cell lung cancer were eligible. Studied dose-levels of Gem Vin, administered on days 1 and 8 every 3 weeks, were (mg m^–2^): level I = 1000/25; level II = 1200/25; level III = 1000/30; level IV = 1200/30. A feasibility study was performed at each dose-level, followed by a single-stage phase II study. Dose-level IV was unfeasible because of grade 4 neutropenia. Overall, out of 126 patients enrolled in phase II studies, there were one complete and 32 partial responses (response rate 26%: 95% CI 18–34%). Response rates were 27.9%, 21.4% and 29.3% at levels I, II and III, respectively. The treatment was well tolerated. Toxicity was less frequent and severe at level I. Overall median survival was 33 weeks (95% CI 28–40). Descriptive quality of life analysis showed that patients with a worse baseline global health status score tended to drop out of the study earlier than those with a better score. Gem Vin is feasible at different doses. It is sufficiently active and well tolerated. A phase III study to compare the effect on quality of life of Gem Vin (level I) vs cisplatin-based chemotherapy is ongoing. © 2000 Cancer Research Campaign


British Journal of Cancer (2000) 83(6), 707–714
© 2000 Cancer Research Campaign
doi: 10.1054/ bjoc.2000.1341, available online at http://www.idealibrary.com on IDEAL®

### Gemcitabine plus vinorelbine in advanced non-small cell lung cancer: a phase II study of three different doses

**C Gridelli[1], L Frontini, F Perrone, C Gallo, M Gulisano, S Cigolari, F Castiglione, SF Robbiati, G Gasparini, GP Ianniello, A Farris, MC Locatelli, R Felletti and E Piazza, on behalf of the Gem Vin Investigators**

See Appendix for complete list of Authors and Institutions

**Summary** Our aim was to study the activity and toxicity of the gemcitabine plus vinorelbine (Gem Vin) combination and to identify the optimal dose. Previously untreated patients aged < 70 years, with stage IV or IIIb (not candidates for radiotherapy) non-small cell lung cancer were eligible. Studied dose-levels of Gem Vin, administered on days 1 and 8 every 3 weeks, were (mg $m^{-2}$): level I = 1000/25; level II = 1200/25; level III = 1000/30; level IV = 1200/30. A feasibility study was performed at each dose-level, followed by a single-stage phase II study. Dose-level IV was unfeasible because of grade 4 neutropenia. Overall, out of 126 patients enrolled in phase II studies, there were one complete and 32 partial responses (response rate 26%: 95% CI 18–34%). Response rates were 27.9%, 21.4% and 29.3% at levels I, II and III, respectively. The treatment was well tolerated. Toxicity was less frequent and severe at level I. Overall median survival was 33 weeks (95% CI 28–40). Descriptive quality of life analysis showed that patients with a worse baseline global health status score tended to drop out of the study earlier than those with a better score. Gem Vin is feasible at different doses. It is sufficiently active and well tolerated. A phase III study to compare the effect on quality of life of Gem Vin (level I) vs cisplatin-based chemotherapy is ongoing. © 2000 Cancer Research Campaign

**Keywords**: gemcitabine; vinorelbine; non-small cell lung cancer

Cisplatin-based chemotherapy has been the established tool for palliative treatment of advanced non-small cell lung cancer (NSCLC) patients. A retrospective analysis of trials conducted by the Southwest Oncology Group (Albein et al, 1991) and a meta-analysis showed that cisplatin-based chemotherapy significantly improved survival as compared with the best supportive care (NSCLC Collaborative Group, 1995). However, cisplatin is associated with side-effects that, in some cases, are not easily managed and that probably negatively affect patients' quality of life. This fact is particularly important, because chemotherapy cannot cure advanced NSCLC and its major objective is palliation (Ruckdeshel et al, 1998).

In the last few years, new active drugs have been introduced into the treatment of NSCLC, i.e. vinorelbine, gemcitabine, paclitaxel, docetaxel and irinotecan. Such new drugs are being combined, mostly empirically, in an attempt to identify active, low-toxic, non-cisplatin-containing regimens. Among the possible combinations, gemcitabine plus vinorelbine (Gem Vin) is particularly promising due to the low toxicity of both drugs and to their efficacy as single agents in randomized clinical trials (Depierre et al, 1994; LeChevalier et al, 1994; Manegold et al, 1997; Perng et al, 1997; Sandler et al, 1998; ELVIS Group, 1999).

The aim of the present study was to evaluate toxicity and activity of the Gem Vin combination at four planned dose-levels in order to select the optimal combination to be compared with cisplatin-based chemotherapy in a subsequent randomized trial.

*Received 6 January 2000*
*Revised 10 May 2000*
*Accepted 17 May 2000*

*Correspondence to:* C Gridelli

### PATIENTS AND METHODS

### Eligibility criteria

Patients with good performance status (Eastern Cooperative Oncology Group (ECOG) ≤ 2), below 70 years-of-age, with stage IV or IIIb (with pleural effusion or metastatic supraclavicular lymph nodes) NSCLC were eligible. The diagnosis of NSCLC had to be cytologically or histologically confirmed. Exclusion criteria were the presence of brain metastaes, previous chemotherapy, a history of another cancer (excluding non-melanomatous skin cancer and in situ cervical cancer), reduced bone marrow or renal or hepatic function, and refusal of informed consent. The protocol was approved by the ethics committees of the participating institutions. Patients without measurable disease could be entered in the feasibility part of the study (see below), while they were not eligible for the phase II evaluation.

### Treatment

Gemcitabine and vinorelbine were both given intravenously on days 1 and 8 of a 21-day cycle. Dose-levels were as follows: gemcitabine 1000 mg $m^{-2}$ + vinorelbine 25 mg $m^{-2}$ (level I), gemcitabine 1200 mg $m^{-2}$ + vinorelbine 25 mg $m^{-2}$ (level II), gemcitabine 1000 mg $m^{-2}$ + vinorelbine 30 mg $m^{-2}$ (level III) and gemcitabine 1200 mg $m^{-2}$ + vinorelbine 30 mg $m^{-2}$ (level IV). At an dose-level, treatment was administered for a maximum of six cycles. Treatment was interrupted earlier if progression of the disease was observed. At days 1 and 8, the minimum requirements for chemotherapy were a neutrophil count of $1.5 \times 10^9$ $l^{-1}$ or more, a platelet count of $100 \times 10^9$ $L^{-1}$ or more, a haemoglobin level of 8.0 g $dL^{-1}$ or more, and no sign of organ toxicity (excluding alopecia). If one or more requirements were absent at day 1, chemotherapy was postponed for up to 2 weeks, after which investigators were free to choose the treatment strategy. Chemotherapy was omitted on day 8 if the required haematological parameters were not met. Toxicity and the patient's refusal also resulted in discontinuation of treatment. Antiemetic treatment was provided with standard-dose 5-HT3 antagonists given before chemotherapy administration. Palliative radiotherapy could be delivered, if needed; however, the protocol suggested that contemporaneous chemotherapy and radiotherapy be avoided because of the risk of cumulative toxicity. No second-line treatment was planned by protocol. No prophylactic use of haemotopoietic colony-stimulating factors was planned.

### Staging and follow-up procedures

Before entering the study, all patients underwent a clinical examination that included a PA and lateral chest radiographs, computed tomography of the thorax and abdomen, and a bone scintigram for assessment of disease extension. Before each cycle, patients underwent a clinical examination and a routine biochemistry evaluation that included AST, ALT, bilirubin, alkaline phosphatase, LDH, creatinine, BUN, glucose, uric acid, electrolytes, urine examination and complete blood count. At baseline and after the third and sixth cycles an ECG was performed. Restaging was planned at the end of the third and sixth cycles.

### Toxicity and response evaluation

World Health Organization (WHO) criteria (Miller et al, 1981) were used to categorize toxicity, and the worst degree of toxicity experienced throughout the treatment was computed for each patient.

Response was evaluated at the end of the third and sixth cycles of treatment by repeating staging procedures. The best response was recorded for each patient. For clinically evident or suspected progression of the disease, response evaluation was anticipated. Complete response was defined as the disappearance of all known sites of disease. Partial response was defined as decrease of 50% or more in the sum of products of the largest perpendicular diameters of measurable lesions, with no appearance of new lesions and no progression of any lesion. Stable disease was defined as a decrease of less than 50% or an increase of less than 25% in the sum of products of the largest perpendicular diameters of measurable lesions with no appearance of any new lesion. Progressive disease was defined as a 25% or more increase in the size of one or more measurable lesions, or the appearance of a new lesion. Confirmation of a response after 1 month was not performed. The objective response rate was defined as the proportion of complete and partial responses.

### Quality of life assessment

The European Organization for Research and Treatment of Cancer (EORTC) core questionnaire (QLQ-C30) and lung-cancer-specific module (QLQ-LC13) were used for quality of life (QoL) evaluation. A baseline assessment was planned in the phase II study before each cycle of chemotherapy. The EORTC QLQ-C30 questionnaire consists of multi-item functioning scales and both multi- and single-item scales for the evaluation of general cancer-related symptoms (Aaronson et al, 1993). The EORTC QLQ-LC13 module consists of single items that evaluate specific symptoms of lung cancer (Bergman et al, 1994). Due to the non-formally comparative study design, QoL analysis was only descriptive. The data reported in this paper are only related to the global health status score (items 29 and 30) and are focused on the description of the relationship between mean scores and the overall number of questionnaires completed. A higher value score reflects a better level of quality of life.

### Study design

Because a formal phase I study of the combination was not yet published at the time of protocol writing, a feasibility phase was planned at each of the four dose-levels. Feasibility was performed by one centre (NCI, Naples, Italy) while the phase II multicenter recruitment sequentially started at each dose level immediately after feasibility of that level had been assessed. It was planned that after completion of feasibility evaluation, further assignment of patients to the different dose levels would continue by randomization.

For the feasibility evaluation of the four dose-levels, a criterion analogous to classical phase I studies was applied and cohorts of three patients were enrolled. If no patient out of three, or less than three out of six patients, experienced unacceptable toxicity by the third cycle, that level was considered feasible and the multicenter phase II study at that dose could start. Unacceptable toxicity was defined as any of the following toxic events occurring during any of the first three cycles: grade 4 leukopenia or neutropenia or febrile neutropenia (fever > 38°C with neutrophils < 1000 $mm^3$), grade 3–4 thrombocytopenia or vomiting or mucositis or neurotoxicity, and grade ≥ 2 organ toxicity (except hair loss).

The phase II studies, separately at each feasible dose-level, were planned according to a single-arm design with $p_0 = 0.15$, $p_1 = 0.30$, $\alpha = 5\%$, $\beta = 20\%$. The planned number of patients to be recruited was 42 for each study; 10 objective responses were required in order to define a dose-level active enough to warrant further studies. Recruitment of patients at the different dose-levels was made sequentially (level 1 → level II → level III); immediately after the end of the feasibility study, assignment of patients to the three feasible dose-levels was done by a randomization procedure, with a computer-driven minimization technique that accounted for centre, stage and PS as stratifying variables.

### Statistical analysis

No formal statistical comparisons among dose-levels were performed. For response rates and unacceptable toxicity rates, 95% exact confidence intervals were reported. Time-to-progression (TTP) was defined as the interval elapsed between date of recruitment and the date of ascertained tumour progression or of death without evidence of disease progression. Survival was defined as the time elapsed between the date of recruitment and the date of death or the date of the last follow-up visit. Time-to-progression and survival curves were estimated by the Kaplan–Meier product limit method (Kaplan and Meier, 1958).

### RESULTS

### Feasibility

From February 1997 to February 1998, 15 patients entered the study; their characteristics are reported in column 1 of Table 1.

© 2000 Cancer Research Campaign

**Table 1** Characteristics of patients (percentages in brackets)

| Variable | Feasibility | Phase II | | | |
|---|---|---|---|---|---|
| | | level I | level II | level III | total |
| | (*n* = 15) | (*n* = 43) | (*n* = 42) | (*n* = 41) | (*n* = 126) |
| Centre by number of pts | | | | | |
| ≥ 5 pts | 15 (100) | 33 (76.7) | 34 (81.0) | 37 (90.2) | 104 (82.5) |
| < 5 pts | – | 10 (23.3) | 8 (19.0) | 4 (9.8) | 22 (17.5) |
| Sex | | | | | |
| males | 12 (80.0) | 38 (88.4) | 38 (90.5) | 35 (85.4) | 111 (88.1) |
| females | 3 (20.0) | 5 (11.6) | 4 (9.5) | 6 (14.6) | 15 (11.9) |
| Age | | | | | |
| median | 59 | 62 | 63 | 63 | 63 |
| range | 38–68 | 46–69 | 46–69 | 49–69 | 46–69 |
| ECOG PS | | | | | |
| 0 | 2 (13.3) | 15 (34.9) | 13 (31.0) | 14 (34.2) | 42 (33.3) |
| 1 | 11 (73.3) | 20 (46.5) | 21 (50.0) | 21 (51.2) | 62 (49.2) |
| 2 | 2 (13.3) | 8 (18.6) | 8 (19.0) | 6 (14.6) | 22 (17.5) |
| Stage | | | | | |
| IIIb | 1 (6.7) | 10 (23.3) | 10 (23.8) | 11 (26.8) | 31 (24.6) |
| IV | 14 (93.3) | 33 (76.7) | 32 (76.2) | 30 (73.2) | 95 (75.4) |
| Histology | | | | | |
| squamous | 7 (46.7) | 14 (32.6) | 16 (38.1) | 20 (48.8) | 50 (39.7) |
| adenocarcinoma | 8 (53.3) | 21 (48.8) | 17 (40.5) | 17 (41.5) | 55 (43.7) |
| large cells | – | 1 (2.3) | 2 (4.8) | – | 3 (2.4) |
| not defined | – | 7 (16.3) | 7 (16.7) | 3 (7.3) | 17 (13.5) |
| mixed | – | – | – | 1 (2.4) | 1 (0.8) |
| Weight loss (three missing) | | | | | |
| none | 12 (80.0) | 27 (65.9) | 23 (54.8) | 19 (47.5) | 69 (56.1) |
| ≤ 10% | – | 11 (26.8) | 17 (40.5) | 18 (45) | 46 (37.4) |
| >10% | 3 (20.0) | 3 (7.3) | 2 (4.8) | 3 (7.5) | 8 (6.5) |
| No. of tumour sites | | | | | |
| 0 | 1 (6.7) | – | – | – | – |
| 1 | 2 (13.3) | 3 (7.0) | 5 (11.9) | 4 (9.8) | 12 (9.5) |
| 2 | 6 (40.0) | 17 (39.5) | 14 (33.3) | 12 (29.3) | 43 (34.1) |
| 3 | 6 (40.0) | 11 (25.6) | 14 (33.3) | 16 (39.0) | 41 (32.5) |
| 4 | – | 10 (23.3) | 7 (16.7) | 6 (14.6) | 23 (18.3) |
| 5 | – | 1 (2.3) | 1 (2.4) | 3 (7.3) | 5 (4.0) |
| 6 | – | – | 1 (2.4) | – | 1 (0.8) |
| 7 | – | 1 (2.3) | – | – | 1 (0.8) |

Median age was 59 years (range 38–68). They were predominantly males (80%), with stage IV disease (93.3%), and with a relatively good PS (ECOG 1 in 73.3%). No unacceptable toxicity was observed in the cohorts of three patients treated at level I and at level III. At level II, one patient out of six had grade 2 neutropenia preventing treatment on day 8 and grade 3 peripheral neuropathy. At level IV, the first three patients all suffered grade 4 neutropenia. Thus, levels I, II and III were considered feasible, while level IV was considered unfeasible.

### Phase II studies

From May 1997 to November 1998, 128 patients were enrolled from 20 participating centres. Nine centres enrolling five or more patients accounted for 82.5% of the total number of patients entered. Dose-level was assigned sequentially to the first 75 patients and by minimization to the remaining 53. After registration, one patient, at level III, was excluded because he refused chemotherapy and another, at level II, was excluded because chemotherapy had been started before registration. Thus, 43, 42 and 41 patients were evaluated at levels I, II and III respectively. The main characteristics of the patients were similar in the three groups (Table 1). Overall, median age was 63 years (range 46–69); males were prevalent (88.1%); most patients were well-performing with PS 0 in one-third and PS 1 in half of the sample; 75.4% of the patients had stage IV disease. More than the half of the patients had three or more sites of disease.

Compliance of patients was good. The rate of patients receiving three or more cycles was 69.0% overall, without variations according to dose-level (69.8%, 69.0% and 68.3% at levels I, II and III, respectively). Considering the first three cycles, 76.2% of patients regularly received chemotherapy on day 8; day-8 treatment was withdrawn once in 19.0%, twice in 3.2% and three times in 1.6%. Again, there was no dose-level effect on compliance to day 8 treatment.

Treatment toxicity was mild with no death at any dose-level. Overall, unacceptable toxicity, as defined above (with the exclusion of criteria related to treatment delay that have been described in the compliance paragraph), occurred in 19.8% (95% CI 12.8–26.8%). Higher rates of unacceptable toxicity were observed with higher dose-levels: 16.3%, 19.0% and 24.4% at levels I, Ii and III, respectively. Severe haematological toxicities (Table 2) were grade 4 non-symptomatic thrombocytopenia (one case at each level) and grade 4 anaemia (one case at level III). No febrile neutropenia was recorded. Seven patients (5.6%) required blood transfusions (two, one and four at the three levels, respectively). Fatigue was the most common non-haematological toxicity (Table 3), and was severe in 8.7% of patients. Grade 4 constipation and

© 2000 Cancer Research Campaign

**Table 2** Haematological toxicity (percentages in brackets)

| Toxicity | level I (*n* = 43) | level II (*n* = 42) | level III (*n* = 41) | total (*n* = 126) |
|---|---|---|---|---|
| Leukopenia | | | | |
| grade 3 | 1 (2.3) | 7 (16.7) | 7 (17.1) | 15 (11.9) |
| grade 4 | 2 (4.7) | 2 (4.8) | 4 (9.8) | 8 (6.3) |
| Neutropenia | | | | |
| grade 3 | 4 (9.3) | 10 (23.8) | 5 (12.2) | 19 (15.1) |
| grade 4 | 2 (4.7) | 6 (14.3) | 7 (17.1) | 15 (11.9) |
| Infections | | | | |
| grade 2 | – | – | 1 (2.4) | 1 (0.8) |
| grade 3 | – | 3 (7.1) | 1 (2.4) | 4 (3.2) |
| Thrombocytopenia | | | | |
| grade 3 | – | 2 (4.8) | – | 2 (1.6) |
| grade 4 | 1 (2.3) | 1 (2.4) | 1 (2.4) | 3 (2.4) |
| Bleeding | | | | |
| grade 1 | – | 1 (2.4) | – | 1 (0.8) |
| Anaemia | | | | |
| grade 3 | – | 1 (2.4) | 2 (4.9) | 3 (2.4) |
| grade 4 | – | – | 1 (2.4) | 1 (0.8) |
| Fever | | | | |
| grade 2 | 6 (14.0) | 6 (14.3) | – | 12 (9.5) |
| grade 3 | – | 1 (2.4) | 1 (2.4) | 2 (1.6) |

**Table 3** Non-haematological toxicity (percentage in brackets)

| Toxicity | level I (*n* = 43) | level II (*n* = 42) | level III (*n* = 41) | total (*n* = 126) |
|---|---|---|---|---|
| Fatigue | | | | |
| grade 3 | 2 (4.7) | 1,5 (11.9) | 4 (9.8) | 11 (8.7) |
| Vomiting | | | | |
| grade 3 | – | 1 (2.4) | 1 (2.4) | 2 (1.6) |
| Diarrhoea | | | | |
| grade 2 | – | 2 (4.8) | – | 2 (1.6) |
| grade 3 | 1 (2.3) | – | – | 1 (0.8) |
| Mucositis | | | | |
| grade 2 | 2 (4.7) | – | 1 (2.4) | 3 (2.4) |
| grade 4 | – | – | 1 (2.4) | 1 (0.8) |
| Constipation | | | | |
| grade 2 | 4 (9.3) | 2 (4.8) | – | 6 (4.8) |
| grade 4 | – | – | 1 (2.4) | 1 (0.8) |
| Neuropathy | | | | |
| grade 3 | 1 (2.3) | 1 (2.4) | – | 2 (1.6) |
| CNS | | | | |
| grade 1 | 3 (7.0) | – | 1 (2.4) | 4 (3.2) |
| Cutaneous | | | | |
| grade 2 | 1 (2.3) | 1 (2.4) | 1 (2.4) | 3 (2.4) |
| Heart rhythm | | | | |
| grade 2 | – | – | 1 (2.4) | 1 (0.8) |
| Pulmonary | | | | |
| grade 2 | 1 (2.3) | 1 (2.4) | – | 2 (1.6) |
| grade 3 | – | 1 (2.4) | – | 1 (0.8) |
| Liver | | | | |
| grade 2 | 1 (2.3) | 1 (2.4) | – | 2 (1.6) |
| grade 3 | – | 1 (2.4) | 1 (2.4) | 2 (1.6) |
| grade 4 | 1 (2.3) | – | – | 1 (0.8) |
| Bone pain | | | | |
| grade 2 | 1 (2.3) | 2 (4.8) | 1 (2.4) | 4 (3.2) |
| grade 4 | 1 (2.3) | – | – | 1 (0.8) |
| Hair loss | | | | |
| grade 2 | 1 (2.3) | 2 (4.8) | 4 (9.8) | 7 (5.6) |

grade 4 mucositis were recorded in one case each, both at level III. Liver toxicity, measured by increase of AST and ALT, was reported at level I in two cases (one grade 2 and one grade 4), at level II in two other cases (one grade 2 and one grade 3) and at level III in one case only (grade 3). Heart rhythm disturbances, possibly related to treatment, were reported in one patient, at level III.

© 2000 Cancer Research Campaign

**Table 4** Treatment activity (percentages in brackets)

| Response | level I (*n* = 43) | level II (*n* = 42) | level III (*n* = 41) | total (*n* = 126) |
|---|---|---|---|---|
| Complete | – | – | 1 (2.4) | 1 (0.8) |
| Partial | 12 (27.9) | 9 (21.4) | 11 (26.8) | 32 (25.4) |
| Response rate | 27.9% | 21.4% | 29.3% | 26.2% |
| 95% exact CI | 15.3–43.7 | 10.3–36.8 | 16.1–45.5 | 18.5–33.9 |
| Stable disease | 11 (25.6) | 11 (26.2) | 7 (17.1) | 29 (23.0) |
| Progression | 15 (34.9) | 19 (45.2) | 14 (34.1) | 48 (38.1) |
| Not evaluated | 5 (11.6) | 3 (7.1) | 8 (19.5) | 16 (12.7) |

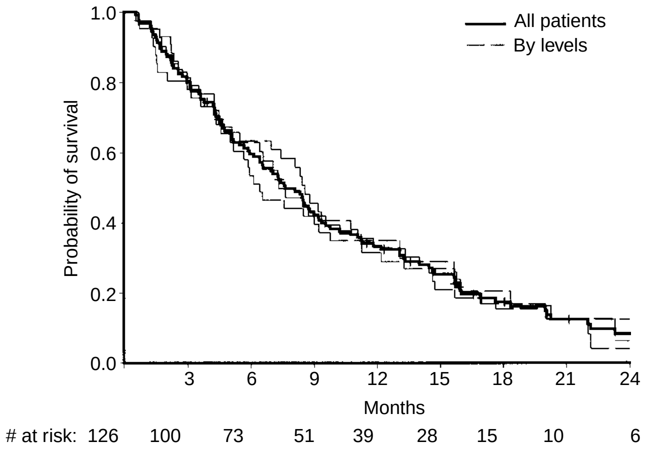

**Figure 1** Overall survival curves

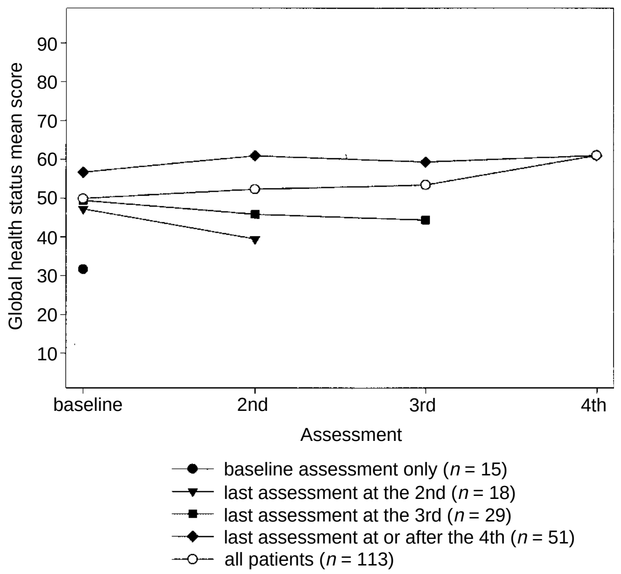

**Figure 2** Global health status mean scores by QoL drop-out timing

Phlebitis was reported in three (2.4%) patients, hair loss was infrequent, reaching grade 2 in seven (5.6%) patients. No renal toxicity was recorded.

Overall, 33 patients (26.2%, 95% CI 18.5–33.9) had an objective response: complete in one case and partial in 32 (Table 4). According to study rules, the minimum expected number of responses was surpassed at levels I (12 partial responses, 27.9%, 95% CI 15.3%–43.7%) and III (one complete response and 11 partial responses, 29.3%, 95%, CI 16.1–45.5), but not at level II (nine partial responses, 21.4%, 95% CI 10.3–36.8). The disease stabilized in 29 (23.0%) patients.

### Survival

As of November 30 1999, 85 patients had had a clinical or radiological evident tumour progression and 107 had died (33 of these, without evidence of disease progression, were considered as progressed on the date of death). Median TTP was 18 weeks (95% CI 14–22); 6-month and 1-year TTP were 33% and 16%, respectively; out of eight patients not progressed, four had a follow-up time longer than 1 year. Median survival was 33 weeks (95% CI 28–40); 6-month, 12-month and 18-month survival being 60%, 33% and 17%, respectively. As shown in Figure 1, overall survival curves of the total study population and those of subgroups treated at different dose-levels are very similar.

### Quality of life

Compliance to QoL questionnaires progressively decreased across the cycles (89.7% at baseline and 69.8%, 59.5%, 35.7%, 28.6%, 26.2% before the second, third, fourth, fifth and sixth cycle, respectively). Figure 2 shows the mean scores of the global health status item for all patients (white symbols) and for subgroups of patients according to the time of the last QoL assessment, combining patients who completed four or more questionnaires. Patients with a lower (worse) baseline global health status score tended to drop out of the study earlier than patients with a higher (better) score. Furthermore, a slight worsening of the score was observed within each subgroup although an improvement was apparent when measuring mean values in the whole group, because of the selection bias due to early drop-out of patients with unfavourable prognosis.

### DISCUSSION

The principal strategic consideration underlying this study was that cisplatin-based chemotherapy only produces a modest survival advantage in advanced NSCLC (NSCLC Collaborative

© 2000 Cancer Research Campaign

Group, 1995), and is frequently associated with toxicity. Thus, we aimed to look for a less toxic but sufficiently active treatment to be subsequently compared in a randomized trial to cisplatin-based chemotherapy.

Knowledge of the efficacy of chemotherapy without cisplatin for advanced NSCLC has for a long time been based on the results of clinical trials performed many years ago. Meta-analysis of trials comparing chemotherapy without cisplatin vs best supportive care yielded negative results. But the trials included in the meta-analysis explored the use of long-term alkylating agents or etoposide as a single agent (NSCLC Collaborative Group, 1995), which are no longer used because of their very low activity.

Clinical trials comparing regimens containing mitomycin C and vindesine (MV) vs cisplatin-based treatment partially modified our perspective. In fact, two randomized trials of MV vs cisplatin-containing treatment yielded contrasting results (Shinkai et al, 1985; Luedke et al, 1990). In another randomized trial comparing MV vs mitomycin C plus ifosfamide vs cisplatin plus etoposide, no significant differences were seen in response rate and survival among the three arms but, on the basis of toxicity, mitomycin C plus vindesine was felt to be the preferred regimen (Gatzmeier et al, 1991). Furthermore, in a phase III trial on 204 metastatic NSCLC patients in which mitomycin C plus etoposide plus vindesine (MEV) was compared with cisplatin plus mitomycin C plus vindesine (MVP), we observed no differences in relief of symptoms, response rate or survival among the two arms, but MEV had a significantly lower toxicity (Gridelli et al, 1996).

The advent of new active and well-tolerated drugs prompted further research in this field, also in the light of the increasing importance of QoL as a major end-point of the treatment of advanced NSCLC.

Gem Vin combination was feasible at three out of four tested dose-levels. At the dose of 1200 mg $m^{-2}$ and 30 mg $m^{-2}$, it was less manageable because of severe neutropenia. As we had no indication that higher doses could improve patient outcome, and because low toxicity was among the main objectives in view of a QoL-based comparison with cisplatin-containing chemotherapy, we explored the activity and toxicity of Gem Vin at all feasible levels. The final choice of the optimal schedule to be compared with cisplatin-containing chemotherapy was qualitatively based on the balance between activity and toxicity. In this view, the first dose-level (gemcitabine 1000 mg $m^{-2}$ + vinorelbine 25 mg $m^{-2}$, days 1 and 8, every 3 weeks) seems to be the optimal choice, being similarly active and less toxic than the other two dose-levels studied.

It has recently been suggested (Chen et al, 1999) that the sequence of administration of the two drugs might affect activity and toxicity of the Gem Vin combination. Chen et al found that giving vinorelbine first produced a higher response rate, as compared to a previous study where gemcitabine was given first. Also, a higher degree of toxicity, mainly myelosuppression, was observed with vinorelbine given first. In our study it was planned to give gemcitabine as first drug. However, 15 patients received the inverse sequence of drugs. Although this kind of analysis was not planned, we checked whether there was any effect on the outcomes of the sequence of drug administration. We found no association between the sequence and response rate (31/111, 28% with Gem→Vin; 2/15, 13% with Vin→Gem; $P$ = 0.23). Instead, the Vin→Gem was more toxic as for leukopenia (chi-square for trend $P$ = 0.03) and neutropenia (chi-square for trend $P$ = 0.0074); namely, 9% of patients had grade 4 neutropenia with Gem→Vin vs 33% with Vin→Gem. There was no other significant difference in toxicity between the two schedules. Thus, our data are contrasting with those of Chen for activity and consistent for toxicity. However, both our analysis and the data of Chen may only be considered as suggestive, due to a number of biases that could affect retrospective analysis and comparisons with historical controls.

An interesting finding emerged from QoL analysis, which we had already previously noted in elderly patients with advanced NSCLC (ELVIS group, 1999). Global health status mean scores, which are reliable indicators of QoL, were closely related to the timing of patients' drop-out. Therefore, calculations made on the whole patient population are clearly misleading and over-optimistic, because a relevant rate of progressions or deaths occur quite early in patients with advanced NSCLC given the natural history of the disease. Consequently, QoL unadjusted for the drop-out process, as is frequently reported in studies on NSCLC, may be misleading and could lead to incorrect conclusions.

To our knowledge, this is the largest and most comprehensive phase II evaluation of a new non-cisplatin-based treatment regimen in advanced NSCLC. As for the combination gemcitabine plus vinorelbine, two phase II trials have recently been reported, with relevant differences as for schedule of treatment or patient selection as compared to the present one. Isokangas et al (1999) explored two different schedules in a group of adult patients with advanced NSCLC, including a subgroup with stage IIIb disease without metastatic supraclavicular nodes or pleural effusion, and including a subgroup of elderly patients (median age 59, age range 40–78). They found that a 28-day cycle with vinorelbine 30 mg $m^{-2}$ on days 1, 8, 15 and 22 and gemcitabine 1000 mg $m^{-2}$ on days 1, 8 and 15 was too toxic, mainly because of severe neutropenia producing frequent delays of drug administration; in addition, three out of 12 patients treated with this schedule died because of toxicity. Further, 32 patients were treated with a fortnightly schedule with vinorelbine 35 mg $m^{-2}$ and gemcitabine 1200 mg $m^{-2}$ both given on days 1 and 15 of each 28-day cycle. The overall response rate (including those patients considered not evaluable by the Authors because they had received less than two cycles) was 40.6% (95% CI 23.7–59.4%, 13/32 patients), possibly favoured some patients with less advanced stage IIIb disease. As for toxicity, the Authors signal that 24% of patients suffered grade 3–4 neutropenia with the fortnightly schedule. Overall, the fortnightly administration, which implies a prolongation of overall treatment duration as compared to the Gem Vin schedule we applied, does not seem to produce better results. Feliu et al (1999) selected 49 patients with advanced NSCLC for treatment with a combination of gemcitabine plus vinorelbine because they were > 70 years old (38/49 patients, 78%) or, if younger, they had some contraindications to receiving cisplatin. They also included 19 patients (39%) with stage IIIb disease eligible for radiotherapy. Vinorelbine 25 mg $m^{-2}$ and gemcitabine 1000 mg $m^{-2}$ were given on days 1, 8 and 15 every 28 days. Toxicity was mild; the overall response rate was 26.5% (95% CI 14.9–41.1%), and median survival was 33 weeks. As for other combinations including new drugs, a phase II trial on 46 patients treated with docetaxel plus vinorelbine plus G-CSF support showed relevant toxicity (four toxic deaths, 24% of patients with neutropenic fever, 43% of patients requiring hospitalization) and a 33% response rate (Kourousis et al, 1998). Another phase II trial of docetaxel plus gemcitabine plus G-CSF (Georgulias et al, 1999) in 51 patients showed a 37% response rate

 *© 2000 Cancer Research Campaign*

with apparently manageable toxicity. It is important, in our opinion, to stress that both the latter two treatment schedules required the administration of supportive G-CSF by principle, with relevant impact on cost of treatment and, possibly, on quality of life.

The study design applied in the present phase II study might look unusual. It was prompted by the consideration that no formal phase I study had been published at the time the protocol was drawn, but the treatment had been sporadically tested in clinical practice for patients not eligible to cisplatin-based chemotherapy, at different doses, by many of the participating investigators. Thus, a short feasibility study, based on a common phase I design, was planned at each dose-level. In addition, investigators agreed initially that all the four dose-levels on study could probably be active against the disease, although with possibly different toxicity rates. Thus, we decided that a phase II trial was worth conducting at all feasible doses. The procedure of minimization, used to randomize 42% of patients on-study, did produce similar distribution of main baseline (possibly prognostic) characteristics of patients among the three dose-levels. Although inter-arm comparisons among dose-levels were not formally applied, the attained sample and the observed results rule out the possibility that large differences of activity may exist among tested doses (Simon et al, 1985). Further, overall results represent a very reliable estimate of treatment activity, due to the large sample size in this phase II study. This is reassuring, in the light that for the proposed phase III study (comparing GemVin vs cisplatin-based chemotherapy) some concern could arise as for the risk of under-treatment with GemVin.

According to the results of the present study, a two-arm phase III study comparing a combination of gemcitabine (1000 mg $m^{-2}$) and vinorelbine (25 mg $m^{-2}$) vs the cisplatin-based chemotherapy currently used in Italy (cisplatin plus gemcitabine or cisplatin plus vinorelbine) is ongoing. The primary end-point of this trial is QoL and an interim survival analysis is planned to protect patients from large survival differences between the two arms.

### ACKNOWLEDGEMENTS

The GemVin Investigators thank Federika Cruolele, Giuliana Canzanella, and Assunta Caiazzo for data management and secretarial services, Alfonso Savio for software assistance and Jean Gilder for editing the text. This paper was presented in part at the 1999 American Society of Clinical Oncology Meeting.

### REFERENCES

Aaronson NK, Ahmedzai S, Bergman B, Bullinger M, Cull A, Duez NJ, Filiberti A, Fletchner H, Fleishman SB, de Haes JCJM, Kaasa S, Klee M, Osoba D, Razavi D, Rofe PB, Schraub S, Sneeuw K, Sullivan M and Takeda F for the EORTC Study Group on Quality of Life (1993) The EORTC QLQ-C30: a quality of life instrument for use in international clinical trials in oncology. *J Natl Cancer Inst* **85**: 365–376

Albein KS, Crowley II, LeBlanc M and Livingston B (1991) Survival determinants in extensive disease non small cell lung cancer: the Southwest Oncology Group experience. *J Clin Oncol* **9**: 1618–1626

Bergman B, Aaronson NK, Ahmedzai S, Kaasa S and Sullivan M for the EORTC Study Group on Quality of Life (1994) The EORTC QLQ-LC13: a modular supplement to the EORTC core quality of life questionnaire (QLQ-C30) for use in lung cancer clinical trials. *Eur J Cancer* **30A**: 635–642

Chen YM, Whang-Peng J, Perng RP, Liu TW, Yang KY, Lin WC, Wu HW, Shih JF, Liu JM, Chen LT and Tsai CM (1999) A multicenter phase II study of gemcitabine and vinorelbine in patients with advanced stage IIIB-IV non small cell lung cancer. *Proc Am Soc Clin Oncol* **18**: 481a, 1856

Depierre A, Chastang C, Quoix E, Lebeau B, Blanchon F, Paillot N, Lemarie E, Hilleron B, Moro D, Clavier J, Herman D, Tuchais E, Jacoulet P, Brechot JM, Cordier JF, Jolal-Celigny P, Badri N and Besenval M (1994) Vinorelbine versus vinorelbine plus cisplatin in advanced non-small-cell lung cancer: a randomized trial. *Ann Oncol* **5**: 37–42

Elderly Lung Cancer Vinorelbine Italian Study (ELVIS) Group (1999) Effects of vinorelbine on quality of life and survival of elderly patients with advanced non-small-cell lung cancer. *J Natl Cancer Inst* **91**: 66–72

Feliu J, Lopez Gomez L, Madronal C, Espinosa E, Espinosa J, Garcia Giron C, Martinez B, Castro J, De la Gandara I and Baron MG for the Oncopaz Cooperative Group (1999) Gemcitabine plus vinorelbine in non small cell lung carcinoma patients age 70 years or older or patients who cannot receive cisplatin. *Cancer* **86**: 1463–1469

Gatzmeier U, Heckmayr M, Hossfeld DK, Kaukel E, Koschel G and Neuhauss R (1991) A randomized trial of mitomycin C/ifosfamide vs mitomycin C/vindesine, vs cisplatin/etoposide in advanced non small cell lung cancer. *Am J Clin Oncol* **14**: 405–411

Georgoulias V, Kouroussis C, Androulakis N, Kakolyris S, Dimopoulos MA, Papadakis E, Bouros D, Apostolopoulou F, Papadimitriou C, Agelidou A, Hatzakis K, Kalbakis K, Kotsakis A, Vardalis N and Vlachonicoli J (1999) Front-line treatment of advanced non-small-cell lung cancer with docetaxel and gemcitabine: a multicenter phase II trial. *J Clin Oncol* **17**: 914–920

Gridelli C, Perrone F, Palmeri S, D'Aprile M, Cognetti F, Rossi A, Gebbia V, Pepe R, Veltri E, Airoma G, Russo A, Incoronato P, Scinto AF, Palazzolo G, Natali M, Leonardi V, Gallo C, De Placido S and Bianco AR (1996) Mitomycin C plus vindesine plus etoposide (MEV) versus mytomicin C plus vindesine plus cisplatin (MVP) in stage IV non small cell lung cancer: a phase III multicenter randomised trial. *Ann Oncol* **7**: 821–826

Kaplan EL and Meier P (1958) Non parametric estimation from incomplete observation. *J Am Stat Assoc* **53**: 457–481

Kourousis C, Androulakis N, Kakalyris S, Souglakos J, Maltezakis G, Metaxaris G, Chalkiadakis G, Samonis G, Vlachonikolis J and Georgoulias V (1998) First-line treatment of advanced non small cell lung carcinoma with docetaxel and vinorelbine. *Cancer* **83**: 2083–2090

Isokangas OP, Knuuttila A, Halme M, Mäntylä M, Lindström I, Nikkanen V, Viren M, Joensuu H and Mattson K (1999) Phase II study of vinorelbine and gemcitabine for inoperable stage IIIB-IV non-small-cell lung cancer. *Ann Oncol* **10**: 1059–1063

LeChevalier T, Brisgand D, Douillard JY, Pusol JL, Alberola V, Monnier A, Riviere A, Lianes P, Chomy P, Cigolari S, Gottfried M, Ruffie P, Panizo A, Gaspard MH, Ravaioli A, Besenval M, Besson F, Martinez A, Berthaud P and Tursz T (1994) Randomized study of vinorelbine and cisplatin versus vindesine and cisplatin versus vinorelbine alone in advanced non-small-cell lung cancer: results of a European multicenter trial including 612 patients. *J Clin Oncol* **12**: 360–367

Luedke DW, Einhorn J, Omura GA, Ravisarma P, Bartolucci AA, Birch R and Greco FA (1990) Randomized comparison of two combination regimens versus minimal chemotherapy in non small cell lung cancer. A Southern Cancer Study Group Trial. *J Clin Oncol* **8**: 886–891

Manegold C, Bergman B, Chemaissani A, Dornoff W, Drings P, Kellokumpu-Lehtinen P, Liippo K, Mattson K, von Pawel J, Ricci S, Sederholm C, Stahel RA, Wagenius G, von Walree N and ten Bokkel-Huinink W (1997) Single-agent gemcitabine versus cisplatin-etoposide: early results of a randomized phase II study in locally advanced or metastatic non-small-cell lung cancer. *Ann Oncol* **8**: 525–529

Miller AB, Hoogstraten B, Staquet M and Winkler A (1981) Reporting results of cancer treatment. *Cancer* **47**: 207–214

Non Small Cell Lung Cancer Collaborative Group (1995) Chemotherapy in non small cell lung cancer: a meta-analysis using updated data on individual patients from 52 randomized trials. *BMJ* **311**: 899–909

Pern RP, Chen YM, Ming-Liu J, Tsai C-M, Lin WC, Yang KY and Whang-Peng J (1997) Gemcitabine versus the combination of cisplatin and etoposide in patients with inoperable non-small-cell lung cancer in a phase II randomized study. *J Clin Oncol* **15**: 2097–2102

Ruckdeshel JC, Gridelli C, Perrone F, Monfardini S and Giaccone G (1998) Are platinum compounds mandatory in the treatment of metastatic non small cell lung cancer? *Eur J Cancer* **13**: 1993–1999

Sandler A, Nemunaitis J, Denham C, Cormier Y, von Pawel J, Niyikiza C, Nguyen B and Einhorn L (1998) Phase III study of cisplatin with or without gemcitabine in patients with advanced non small cell lung cancer. *Proc Am Soc Clin Oncol* **17**: 454a–1747

© 2000 Cancer Research Campaign

Shinkai T, Sajio N, Tominaga K, Eguchi K, Shimizu E, Sasaki Y, Fujita J and Futami H (1985) Comparison of vindesine plus cisplatin or vindesine plus mitomycin in the treatment of advanced non small cell lung cancer. *Cancer Treat Rep* **69**: 945–951

Simon R, Wittes RE and Ellenberg SS (1985) Randomized phase II clinical trials. *Cancer Treat Rep* **69**: 1375–1381

### APPENDIX

### Participating Authors and Institutions

C Gridelli, A Rossi, L Barzelloni, Oncologia Medica B IstitutoNazionale Tumori, Napoli; L Frontini, S Zonato, M Cabiddu, Oncologia Medica Ospedale S. Paolo, Milano; M Gulisano, S Schiavon, V Fosser, Oncologia Medica Ospedale S. Bortolo, Vicenza; S Cigolari, V Angelini, V Guardasole, III Medicina Interna Università Federico II, Napoli; F Castiglione, A Venturino, Oncologia Medica Ospedale Civile, Alba [CN]; S F Robbiati, Day Hospital Oncologico Ospedale Civile, Rovereto [TN]; G Gasparini, A Morabito, Oncologia Medica Ospedali Riuniti, Reggio Calabria; G P Ianniello, M G Caprio, Oncologia Medica Università, Sassari; M C Locatelli, Oncologia Medica Ospedale S. Carlo, Milano; R Felletti, Pneumologia Ospedale S. Martino, Genova; E Piazza, Oncologia Medica Ospedale Sacco, Milano; C Nascimbene, Pneumologia Policlinico S. Matteo, Pavia; S Bretti, Oncologia Medica Ospedale S. Giovanni Antica Sede, Torino; F Carrozza, Medicana Interna Ospedale Cardarelli, Campobasso; F Perrone, Ufficio Spermentazioni Cliniche Controllate, Istituto Nazionale Tumori, Napoli; C Gallo, Metodologia Epidemiologica Clinica, Seconda Università di Napoli, Italia.

### We also thank the following Institutions

Oncologia Medica Ospedale S. Gennaro, Napoli; Radioterapia Oncologica Ospedale S. Gerardo, Monza (MI); Oncologia Medica Policlinico S. Matteo, Pavia; Oncologia Medica Ospedale S. Maria Goretti, Latina; Centro di Riferimento Oncologico Ospedale Giustino Fortunato, Rionero in Vulture (PZ).

 *© 2000 Cancer Research Campaign*
